# A New Type of Self-Compacting Recycled Pervious Concrete Under Sulfate Drying–Wetting Exposure

**DOI:** 10.3390/ma18030704

**Published:** 2025-02-05

**Authors:** Xiancui Yan, Zimo He, Qun Xia, Cen Zhao, Pinghua Zhu, Meirong Zong, Minqi Hua

**Affiliations:** 1 Department of Civil Engineering, Changzhou University, Changzhou 213164, China; yanxc@cczu.edu.cn (X.Y.); 15195385025@163.com (Z.H.); xiaqun@cczu.edu.cn (Q.X.); cenoppa@163.com (C.Z.); zph@cczu.edu.cn (P.Z.); 2 School of Civil Engineering & Architecture, Wuhan University of Technology, Wuhan 430070, China; hmq@whut.edu.cn

**Keywords:** recycled pervious concrete, sulfate attack, drying–wetting exposure regimes, permeability

## Abstract

Traditional pervious concrete poses significant challenges in optimizing both mechanical properties and permeability. To address this issue, a novel type of self-compacting recycled pervious concrete (SCRPC) featuring vertical and penetrating channels has been developed. The vertical channels were created by pulling out the reinforcement in the pre-drilled holes that were artificially created in the mold, after the concrete had been poured. However, whether this concrete has superior durability and can be employed in different sulfate drying–wetting situations remains to be investigated. This study explored the sulfate resistance and permeability of SCRPC under five drying–wetting exposure regimes: full soaking in Na_2_SO_4_ solution with drying–wetting ratios of 3:18, 9:12, and 18:3; semi-soaking in Na_2_SO_4_ solution; and full soaking in MgSO_4_ solution. The results showed that the SCRPC soaked in MgSO_4_ solution suffered the largest compressive strength loss (13.4%) after 150 drying–wetting cycles. Furthermore, as the drying–wetting ratio increased, the sulfate degradation of the SCRPC increased. Despite the comparable relative dynamic modulus of elasticity of SCRPC after full soaking (95.54%) and semi-soaking (92.89%), ettringite and gypsum were identified as the predominant sulfate deterioration products of SCRPC, respectively. In contrast to the two stages for traditional pervious concrete, the effective porosity of SCRPC was divided into three stages during sulfate attack: an initial rapid decline stage, a subsequent increase stage, and a final slow decline stage. The permeability coefficient of SCRPC varied from 6.00 to 6.82 mm/s under different sulfate drying–wetting exposures. In summary, SCRPC has superior sulfate resistance and permeability, and it could be more applicable in environments containing Na_2_SO_4_ compared to MgSO_4_. This study provides basic data for the enhancement and application of pervious concrete with artificial vertical and penetrating channels.

## 1. Introduction

The global population explosion and rapid urbanization have disrupted the water cycle, leading to a series of crises, including the urban heat island effect and flood disasters [1,2,3]. Pervious concrete, a pavement material popularized in sponge cities, significantly alleviates urban drainage strain and reduces pavement runoff [4,5,6,7]. Traditional pervious concrete is characterized by high porosity and consists of cementitious materials, coarse aggregate, and little to no fine aggregate [8]. However, due to its high porosity, traditional pervious concrete has low strength and can only be used for low-load traffic roads, such as parking lots and secondary roads. Additionally, sediments (such as sand, silt, etc.) and suspended particles (such as dust, debris, etc.) are easily deposited in concrete pores, decreasing the permeability and service life of pervious concrete [9]. To address the shortcoming of traditional pervious concrete, Li et al. [10] proposed an innovative high-strength pervious concrete by artificial pore-forming. The permeability coefficients of the innovative pervious concrete were found to range from 13.02 mm/s to 21.84 mm/s, with a strength corresponding to 61.37–50.79 MPa. Zhao et al. [11] developed a compressive strength calculation model for innovative high-strength pervious concrete based on the number and diameter of artificial pore distributions. The compressive strength of the concrete decreased as the number and diameter of artificial pores increased. Thus, it is worthwhile to explore the performance of innovative pervious concrete for a wide range of applications.

In recent years, land salinization has become a significant issue in northwest China, resulting in a huge quantity of corrosive ions in soil and sewage [12]. Sulfate is one of the most prevalent corrosive ions, resulting in severe internal expansion damage and strength reduction in concrete [13,14]. Liu et al. [15] pointed out that the main products of Na_2_SO_4_ attack were claviform ettringite and layered gypsum, which have dual effects on the performance of concrete. At present, a number of researchers have investigated the impact of varying exposure regimens on the behaviors of concretes when subjected to sulfate attack. Xie et al. [16] found that concrete semi-soaked in a Na_2_SO_4_ solution exhibited more severe damage than fully soaked concrete, which was attributed to the combined chemical and physical attack. This conclusion has been corroborated by several other studies [17,18]. Zhang et al. [19] demonstrated that drying–wetting cycles accelerated the entry and accumulation of sulfate ions, thereby increasing sulfate absorption near the surface. Yuan et al. [20] considered that drying–wetting cycles aggravated the damage to the internal structure of concrete pores. Additionally, Wang et al. [21] designed four different drying–wetting ratios to study concrete deterioration under sulfate erosion. It was found that the damage caused to the concrete by the drying process occurred in the middle and late periods of erosion. Overall, drying–wetting cycles undoubtedly intensify the erosion process, leading to more severe concrete degradation.

Pervious concrete, as a road material, is particularly susceptible to sulfate attack by drying–wetting cycles [22,23]. Hua et al. [24] indicates that the strength development of pervious concrete under sulfate attack was increasing slowly at first and then decreasing gradually with the number of drying–wetting cycles. The surface damage observed in pervious concrete under sulfate drying–wetting cycles primarily manifests as aggregate peeling, with the peeling process typically occurring from the corners to the diagonals and finally to the sides [25]. In addition to the deterioration of mechanical properties and apparent characteristics of pervious concrete, the permeability performance of pervious concrete could also be impacted by sulfate attack [26,27]. Therefore, it is crucial to investigate the sulfate corrosion resistance and water permeability of pervious concrete during drying–wetting cycles.

Moreover, the failure process of concrete caused by MgSO_4_ is more complex. In a corrosive environment dominated by MgSO_4_, Mg^2+^ replaces Ca^2+^ in C-S-H gel, resulting in the formation of magnesium silicate hydrate (M-S-H) with lower gel strength [28,29], which leads to a loss of concrete strength. Hekal et al. [30] discovered that MgSO_4_ solution caused more damage to concrete than Na_2_SO_4_ solution in terms of chemical corrosion, while the opposite occurred in the case of physical corrosion. Additionally, Jiang and Niu [31] reported that when exposed to drying–wetting cycles, concrete in MgSO_4_ solutions deteriorated more severely than in other sulfate solutions. However, the sulfate resistance and water permeability of pervious concrete in MgSO_4_ solution have received little attention.

China is the world’s largest producer of construction waste, yet its waste concrete recycling rate remains significantly lower than that of developed countries [32]. To utilize waste concrete as much as possible, recycled aggregate was prepared from waste concrete and the performance of the 100% recycled coarse aggregate concrete was evaluated [33]. Based on the concept of Li et al. [10], the combination of 100% recycled coarse aggregate and pervious concrete to produce self-compacting recycled pervious concrete (SCRPC) with artificial pores can effectively resolve the issues of the urban heat island and the disposal of construction waste. This concrete exhibited great durability in fatigue and freeze–thaw environments [34]. However, due to differences in erosion media and drying–wetting cycle regimes, the applicability of SCRPC in different sulfate environments remains questionable. The primary objective of this research is to explore the evolution and behavior of SCRPC in different sulfate drying–wetting environments. Three drying–wetting ratios (18:3, 9:12, and 3:18) and two soaking methods (full soaking and semi-soaking) were used. Two sulfate solutions of 8% Na_2_SO_4_ and 8% MgSO_4_ were selected as erosion media. The mass loss, relative dynamic modulus of elasticity (RDME), and compressive strength of the SCRPC were measured to evaluate its sulfate resistance. In addition, the effective porosity and permeability coefficient of the SCRPC were measured. The deterioration mechanisms were further investigated through analysis of the corrosion products and micromorphology using X-ray diffraction (XRD) and scanning electron microscopy (SEM). This research delves into the potential application of SCRPC in sulfate environments, showing that it still exhibits satisfactory permeability and sulfate resistance and providing a foundation for future engineering applications.

## 2. Materials and Methods

### 2.1. Materials and Mix Proportion of Concrete

Conch brand PO 52.5 ordinary Portland cement, fly ash (FA), and silica fume (SF) were used as the cementitious materials, with apparent densities of 3080, 2520, and 2759 kg/m^3^, respectively. The cement, fly ash (grade II), and silica fume were provided by Jiangsu Yangzi cement plant (Changzhou, China), Changzhou Hutang thermal power plant (Changzhou, China), and Changzhou Dazhenghenggu Building Materials Co., Ltd. (Changzhou, China), respectively. Table 1 shows the chemical compositions of the cementitious materials, as determined by XRF spectroscopy (BRUKER S8 TIGER, Bruker, Bremen, Germany). The cementitious material was ground into a powder with a particle size of less than 200 mesh for XRF testing at an excitation voltage of 60 kV, and the test results were obtained using the QUANT-EXPRESS 4.0 non-standard sample analysis software.

**Figure 1:**
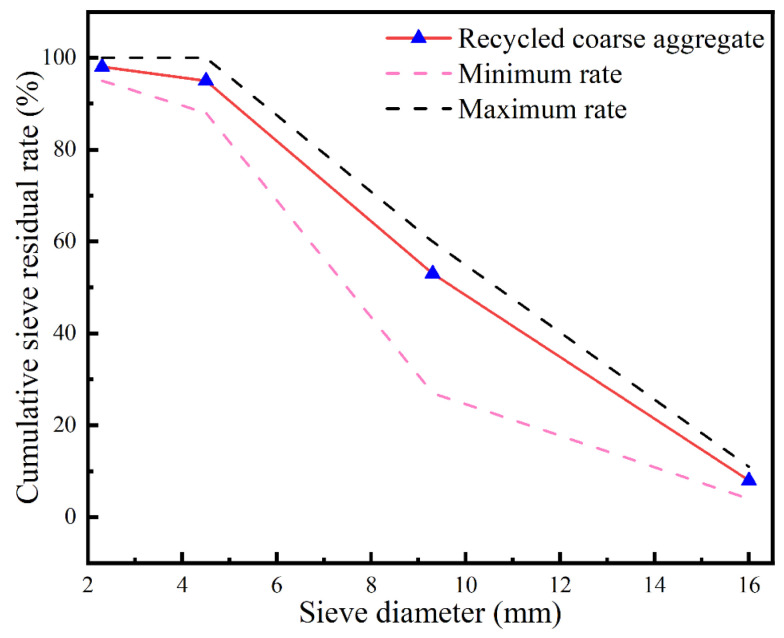
The size distribution of RCA.

Recycled coarse aggregate (RCA) with a particle size of 5–16 mm was supplied by Jiangsu Green River Environmental Technology Company (Changzhou, China). This company is engaged in the recovery, utilization, and production of recycled materials. Figure 1 shows the size distribution of the RCA, which was derived from the sieve analysis results. The fine aggregate used in this study was local natural river sand with a fineness modulus of 2.4 (classified as medium sand), which was provided by Changzhou China Railway Urban Construct Component (Changzhou, China). Polycarboxylic acid superplasticizer (SP) with a water reduction rate of 20% was used as the water-reducing agent. The target strength grade of the concrete was C60, and the mix proportion of the SCRPC is shown in Table 2.

Two sizes of concrete were prepared for this study. Specimens measuring 100 mm × 100 mm × 100 mm were used to measure the compressive strength, effective porosity, and permeability coefficient of the concrete during sulfate exposure; specimens measuring 100 mm × 100 mm × 400 mm were used to measure the RDME and mass loss of the concrete during sulfate exposure.

### 2.2. Mold Design and Specimen Formation

**Figure 2:**
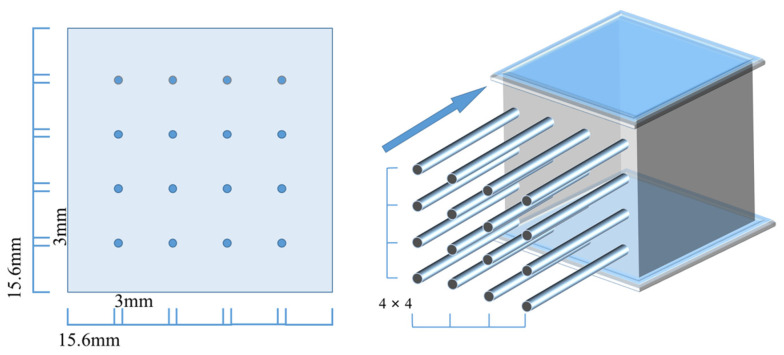
Mold design and assembly of the SCRPC.

**Figure 3:**
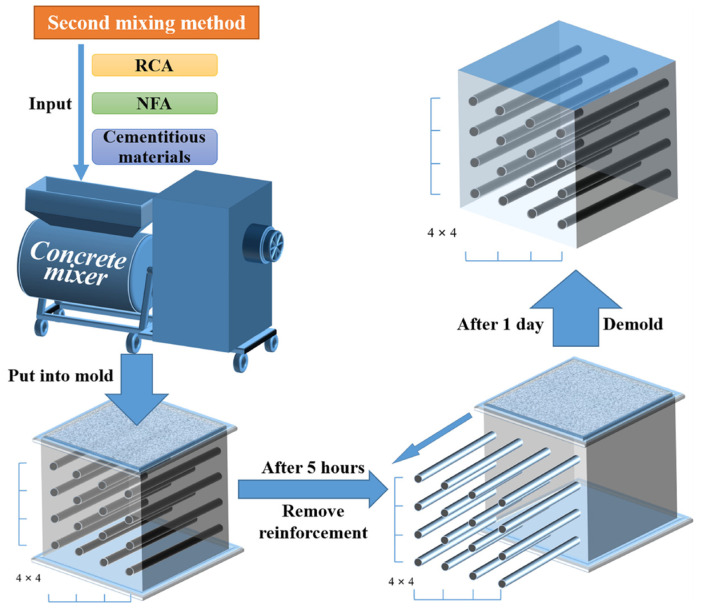
Manufacturing technology of the SCRPC.

To make the SCRPC, molds with reserved holes (3 mm in diameter) were customized. Figure 2 shows the design and assembly of a mold. Before specimen preparation, straight steel bars with a diameter of 3 mm were inserted into the reserved holes in advance. Then, the self-compacting concrete mixture, prepared using the secondary mixing method, was poured into the prepared mold. After 5 h of casting, the straight steel bars were pulled out. It is worth noting that at this time, the mold-forming surface had to be placed sideways in order to prevent the channels from collapsing. After 24 h of casting, the specimen was demolded and immediately placed in a standard curing room for 28 days. The manufacturing technology of the SCRPC was based on recommendations from previous studies in [10,11], as shown in Figure 3.

### 2.3. Drying–Wetting Cycle System

**Figure 4:**
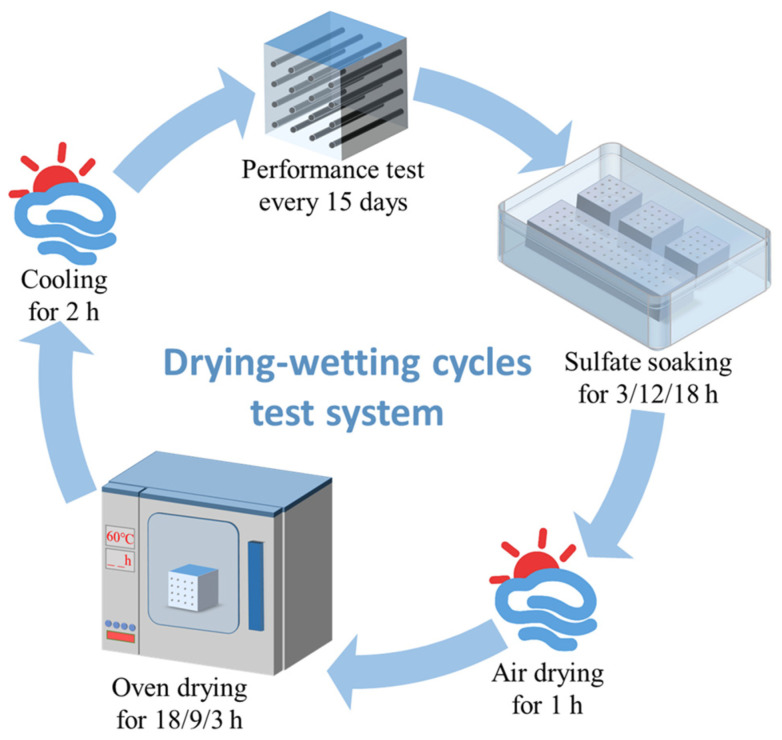
Schematic diagram of drying–wetting cycle system.

After curing, the SCRPC was exposed to different drying–wetting exposure regimes, as shown in Figure 4. Referring to the Chinese standard GB/T50082-2024 [35], the duration of a single drying–wetting cycle was specified as 24 h. A total of 150 drying–wetting cycles were carried out for 150 days. The specific details of the sulfate drying–wetting exposure environment are presented in Table 3.

Exposure 1 (full soaking in 8% Na_2_SO_4_ solution with 3:18 drying–wetting ratio): Each cycle was composed of full soaking in 8% Na_2_SO_4_ solution for 18 h, oven drying at 60 °C for 3 h, and cooling in the air for 3 h.

Exposure 2 (full soaking in 8% Na_2_SO_4_ solution with 18:3 drying–wetting ratio): Each cycle was composed of full soaking in 8% Na_2_SO_4_ solution for 3 h, oven drying at 60 °C for 18 h, and cooling in the air for 3 h.

Exposure 3 (full soaking in 8% Na_2_SO_4_ solution with 9:12 drying–wetting ratio): Each cycle was composed of full soaking in 8% Na_2_SO_4_ solution for 12 h, oven drying at 60 °C for 9 h, and cooling in the air for 3 h.

Exposure 4 (semi-soaking in 8% Na_2_SO_4_ solution with 9:12 drying–wetting ratio): Each cycle was composed of semi-soaking in 8% Na_2_SO_4_ solution for 12 h, oven drying at 60 °C for 9 h, and cooling in the air for 3 h.

Exposure 5 (full soaking in 8% MgSO_4_ solution with 9:12 drying–wetting ratio): Each cycle was composed of full soaking in 8% MgSO_4_ solution for 12 h, oven drying at 60 °C for 9 h, and cooling in the air for 3 h.

### 2.4. Test Methods

The morphology of the specimens under each exposure regime was visually inspected and recorded during the whole test process. The properties (mass loss, RDME, compressive strength, effective porosity, and permeability coefficient) of the SCRPC were measured every 15 days after the specimens were exposed to the corresponding drying–wetting regimes. The test methods of mass loss and RDME refer to the Chinese standard GB/T50082-2024 [35].

#### 2.4.1. Mass Loss

The mass loss of the prismatic specimen was measured by an electronic scale with a precision of 0.1 g. Before the mass measurement, the specimen was air-dried to a constant weight in the laboratory. The mass loss, △*w* (g), was calculated using Equation (1):

(1) $$\Delta w=\frac{w_{t}-w_{0}}{w_{0}}\times 100\%$$

where *w_t_* (g) and *w_0_* (g) are the mass of the specimen before and after *t* times of drying–wetting cycles, respectively.

#### 2.4.2. RDME

The DT-16 dynamic elastic modulus tester was used to detect the fundamental vibration frequency and the dynamic elastic modulus (*E_d_*) of the concrete during sulfate exposure. *E_d_* can be calculated using Equation (2):

(2) $$E_{d}=13.244\times 10^{-4}\times \frac{mL^{3}f^{2}}{a^{4}}$$

where *m* (kg) is the mass of the specimen, *L* (mm) is the total length, *f* (Hz) is the fundamental vibration frequency, and *a* (mm) is the length of the cross-section.

The RDME (*E_rd_*) of concrete during sulfate exposure can be calculated using Equation (3):

(3) $$E_{rd}=\frac{E_{t}-E_{0}}{E_{0}}\times 100\%$$

where *E_0_* (MPa) and *E_t_* (MPa) are the dynamic modulus of elasticity before and after *t* times of drying–wetting cycles, respectively.

#### 2.4.3. Compressive Strength

The compressive strength test method refers to the Chinese standard GB/T 50081–2019 [36]. The loading rate was set as 0.5 MPa/s. The compressive strength, *f_cc_*, was calculated using Equation (4):

(4) $$f_{cc}=\frac{P}{A}$$

where *P* (N) is the peak load and *A* (mm^2^) is the compression area of the concrete.

#### 2.4.4. Effective Porosity

**Figure 5:**
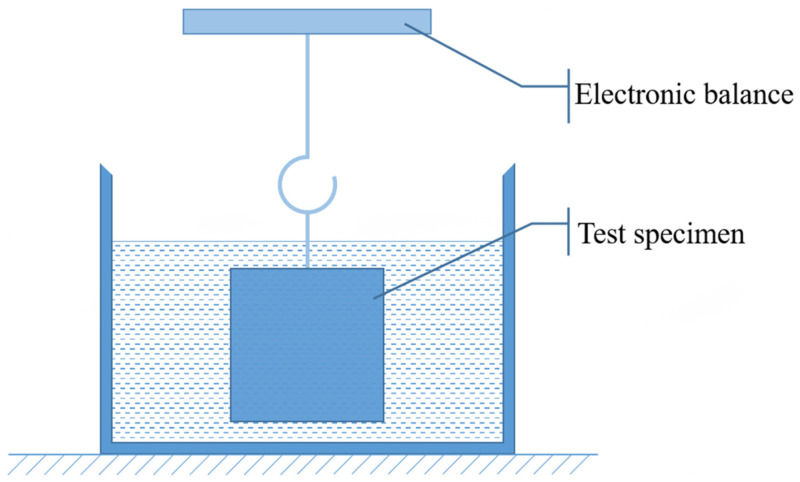
Schematic diagram of SCRPC effective porosity measurement.

The effective porosity of the SCRPC was measured according to the suspension method in Chinese standard CJJ/T 253-2016 [37]. The schematic diagram of the SCRPC effective porosity measurement is shown in Figure 5. The test procedure was as follows. First, the surface of the specimen was cleaned, and the dimensions of the specimen were measured using calipers. Then, the concrete specimen was completely immersed in water and the mass was measured when it stopped bubbling. Finally, the mass of the concrete was measured after drying in an oven at 60 °C for 24 h. The effective porosity of the SCRPC, *P* (%), was calculated using Equation (5):

(5) $$P=(1-\frac{m_{1}-m_{2}}{\rho V})\times 100\%$$

where *m_1_* (g) is the mass of the specimen placed in water, *m_2_* (g) is the mass of the specimen after oven drying at 60 °C for 24 h, *ρ* (g/m^3^) is the density of the water, and *V* (cm^3^) is the volume of the specimen.

#### 2.4.5. Permeability Coefficient

**Figure 6:**
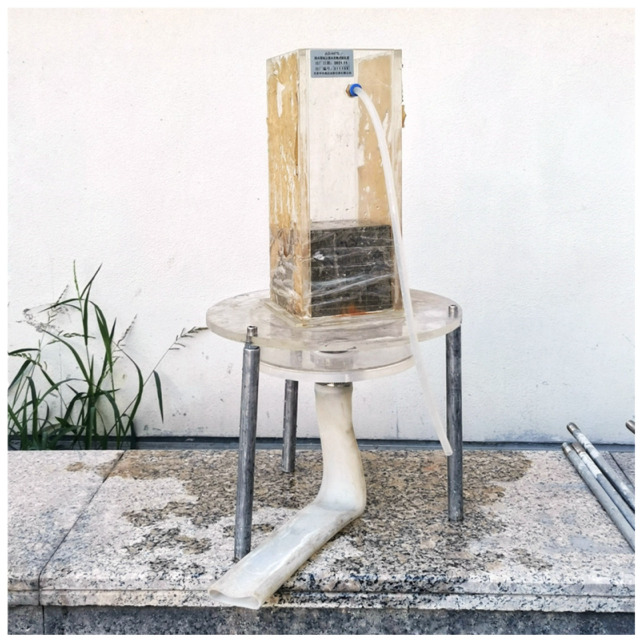
Instrument for determining permeability coefficient.

The constant head method was used to measure the permeability coefficient of the SCRPC according to the Chinese standard CJJ/T 135-2009 [38]. The measuring device of the permeability coefficient is shown in Figure 6. The test procedure was as follows. Firstly, the SCRPC was put into the measuring device. The gaps between the specimen and the walls of the device were filled with butter to make the surroundings watertight. Secondly, water was injected into the device. The surface of the water and the upper surface of the SCRPC were controlled at a certain water level (150 mm). Finally, the water at the outlet of the device was collected and the volume of water was measured within 60 s. The permeability coefficient, *K* (mm/s), was calculated using Equation (6):

(6) $$K=\frac{QL}{AHt}$$

where *Q* (mm^3^) is the volume of water collected within 60 s, *L* (mm) is the thickness of the specimen, *A* (mm^2^) is the upper surface area of the specimen, *H* (mm) is the height difference between the upper head and the lower head, and *T* is 60 s.

#### 2.4.6. Microstructure Properties

After 150 sulfate drying–wetting cycles, the mineral phase and microstructure of the SCRPC were analyzed using an X-ray diffractometer (XRD, D8 ADVANCE, Bruker, Germany) and a scanning electron microscope (SEM, JEOL JSM-6390A, JEOL, Tokyo, Japan), respectively. The samples for XRD analysis were collected within a depth of 5 mm beneath the surface of the straight channel inside the SCRPC. All XRD samples were ground in a bowl and screened with a 0.075 mm sieve. Before the test, the samples were dried for 48 h at a constant temperature of 80 °C. The power samples were measured at a scanning speed of 10° 2θ/min in the 2θ range of 5–90°.

**Figure 7:**
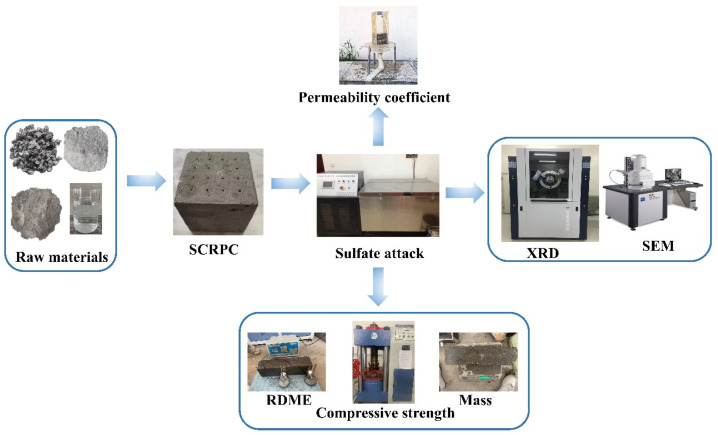
The experimental procedure and process.

For the SEM analysis, representative rectangular samples (5 mm × 5 mm × 2 mm) were collected from the surface of the straight channel inside the SCRPC (0–5 mm near the surface), which is prone to sulfate erosion. After polishing, the samples were sprayed with gold to improve their conductivity before testing. Note that the samples for micro-tests of the semi-soaked specimens were obtained at the upper part of the specimens, above the solution level. Figure 7 depicts the experimental procedure.

## 3. Results

### 3.1. Morphology Change

**Figure 8:**
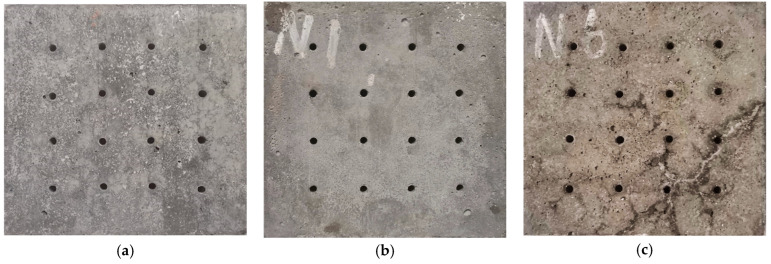

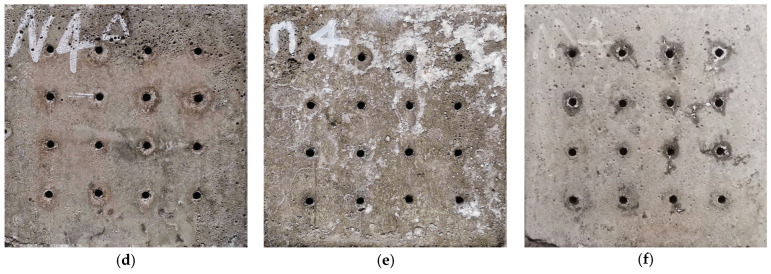
Macroscopic appearance of SCRPC before drying–wetting cycles (**a**) and after 150 sulfate drying–wetting cycles under Exposure 1 (**b**), Exposure 2 (**c**), Exposure 3 (**d**), Exposure 4 (**e**), and Exposure 5 (**f**).

Figure 8 shows the macroscopic appearance of the SCRPC under different sulfate drying–wetting exposure regimes. Before drying–wetting cycles (Figure 8a) or under the Exposure 1 regime (Figure 8b, drying–wetting ratio of 3:18), no cracks were visible on the surface of the specimen. Under the Exposure 2 regime (Figure 8c, drying–wetting ratio of 18:3), a long, thick crack in the lower right corner of the specimen was observed, with some white products. In contrast, the cracks in the specimen were very shallow and short under the Exposure 3 regime (Figure 8d, drying–wetting ratio of 9:12).

It can be also observed that some white products were attached to the surface of the specimen in the semi-soaking exposure regime (Figure 8e, semi-soaking in Na_2_SO_4_ solution). These products filled the pores in the dry area of the upper part of the SCRPC, which may be the crystallization of Na_2_SO_4_ [39,40]. In addition, there were some microcracks around the artificial channel of the cube specimen under the Exposure 5 regime (Figure 8f, 8% MgSO_4_ solution).

### 3.2. Mass Loss

**Figure 9:**
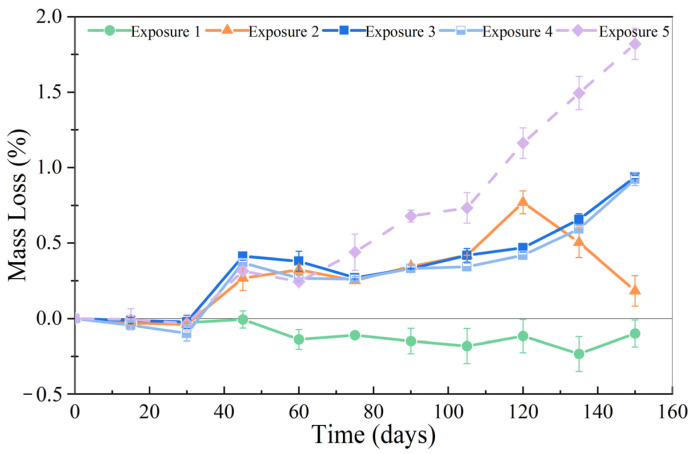
Mass loss of SCRPC specimens under different sulfate drying–wetting exposure regimes.

The mass loss of the SCRPC specimens under different sulfate drying–wetting exposure regimes is shown in Figure 9. During the 150 days of drying–wetting cycles, the mass loss of the SCRPC under Exposure 1 reduced, which was ascribed to the chemical interactions between the cement matrix and sulfate ions that produced gypsum and ettringite. As the number of drying–wetting cycles increased, the mass loss of SCRPC for other exposure regimes first reduced and then increased. Cracks and mortar peeling caused by expansion products brought a negative impact on the mass of the SCRPC. As shown in Figure 9, the SCRPC in 8% MgSO_4_ solution depicted more significant mass loss (1.82%) than that in 8% Na_2_SO_4_ solution (0.94%) after 150 drying–wetting cycles. When the SCRPC was immersed in MgSO_4_ solution, the damage was more severe. The fact that more corrosion products were generated during MgSO_4_ attack than Na_2_SO_4_ attack is consistent with the findings of the earlier investigation [41].

After 150 drying–wetting cycles, the maximum mass loss of SCRPC was observed under Exposure 2 (1.28%), followed by Exposure 3 (0.94%) and Exposure 1 (−0.10%) for full soaking in 8% Na_2_SO_4_ solution. The SCRPC specimen soaked in Na_2_SO_4_ solution with a larger drying–wetting ratio was more likely to suffer greater mass loss. In addition, it was found that the trend of mass variation of SCRPC under the Exposure 4 regime (semi-soaking in Na_2_SO_4_ solution) was similar to that under Exposure 3 (full soaking in Na_2_SO_4_ solution). There is a contradiction regarding the impact of soaking methods on the sulfate deterioration of concrete. For example, Liu’s results [42] indicated that the sulfate deterioration of traditional concrete under semi-soaking could be more serious than that under full soaking. In contrast, Zhang et al. [43] revealed more pronounced damage in the fully immersed condition. Nevertheless, the mass loss of the new type of SCRPC was minimal in a Na_2_SO_4_ environment.

### 3.3. RDME

**Figure 10:**
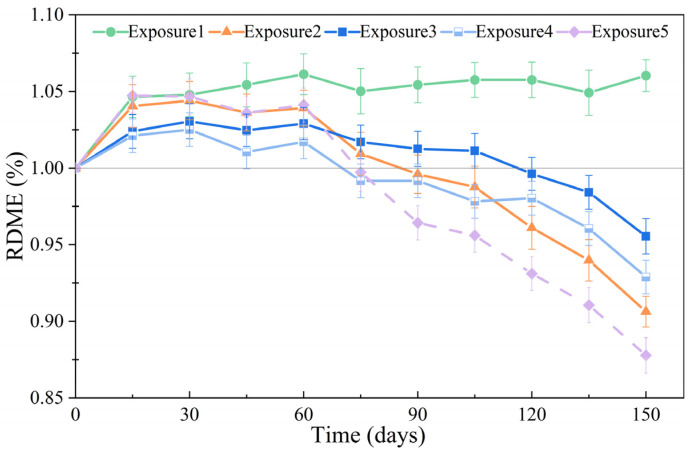
RDME of SCRPC specimens under different sulfate drying–wetting exposure regimes.

Figure 10 shows the RDME of SCRPC specimens under different sulfate drying–wetting exposure regimes. The SCRPC under Exposure 1 (drying–wetting ratio of 3:18) showed almost no deterioration. The RDME of the SCRPC under other exposure regimes was less than 100% after 150 drying–wetting cycles. Further, the RDME values of the SCRPC under Exposure 2 (drying–wetting ratio of 18:3) and Exposure 3 (drying–wetting ratio of 9:12) were 90.63% and 95.54% after 150 cycles, respectively. In the case of sulfate drying–wetting cycles, the production of expansive products and the crystallization pressure of sulfate crystals were the primary causes of concrete degradation. During the wetting stage, the invading sulfate ion reacts with the hydration products, forming expanding degradation products while causing almost no damage due to the micropores formed at the start of the sulfate drying–wetting cycle [21]. During the drying stage, the supersaturated sulfate crystal produces crystallization pressure on the capillary wall, which is more damaging than the expansive products [44]. Thus, a smaller drying–wetting ratio could be beneficial in delaying the damage of SCRPC.

After 150 drying–wetting cycles, the specimen soaking in 8% MgSO_4_ solution displayed the lowest RDME. For the SCRPC in MgSO_4_ solution, the invading sulfate ions reacted with Ca(OH)_2_ and calcium aluminate hydrate to form ettringite and gypsum. Furthermore, the invading Mg^2+^ reacted with C-S-H gel to form M-S-H, which was more destructive than the formation of ettringite and gypsum.

In addition, the RDME of the SCRPC was 95.54% and 92.89% after 150 cycles of full soaking and semi-soaking, respectively. In the case of concrete exposed to sulfate drying–wetting cycles, the main cause of deterioration was the salt crystallization pressure. As the concrete was subjected to semi-soaking in a Na_2_SO_4_ solution, the crystallization pressure increased. Nevertheless, the RDME of the SCRPC is comparable to that of ordinary concrete (with values of 95% after sulfate attack) in [45], demonstrating the superior sulfate resistance of the SCRPC despite the application of 100% recycled coarse aggregate.

### 3.4. Compressive Strength

**Figure 11:**
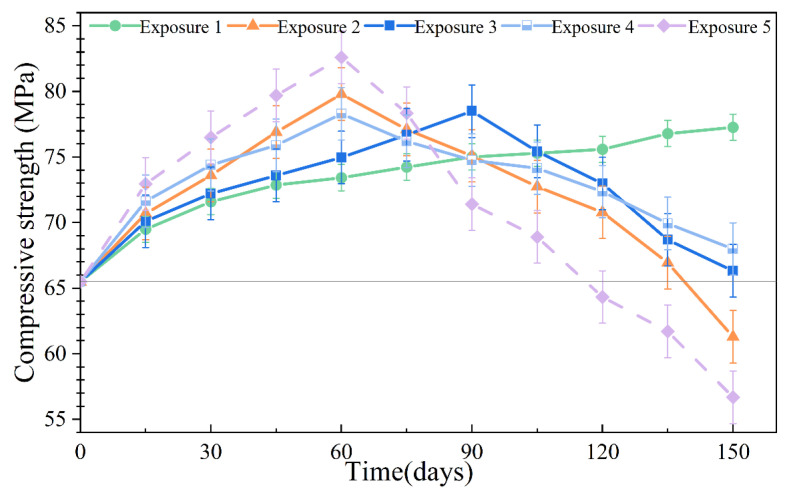
Compressive strength of SCRPC specimens under different sulfate drying–wetting exposure regimes.

The compressive strength of the SCRPC specimens under different sulfate drying–wetting exposure regimes is shown in Figure 11. The compressive strength variation of the SCRPC under drying–wetting cycles consists of two stages: an initial increasing stage and a later decreasing stage, which is similar to previous results [46,47]. Prior to sulfate immersion, the compressive strength of the SCRPC was 65.5 MPa, achieving the target strength. After 150 drying–wetting cycles, the lowest compressive strength occurred in SCRPC fully soaked in 8% MgSO_4_ solution (Exposure 5, 56.7 MPa), with the largest mass loss of 13.4%. Compared to the conventional pervious concrete in [48], which experienced a 26.1% strength reduction after 140 days of erosion, the SCRPC in this paper demonstrated significantly higher resistance to sulfate attack.

The SCRPC fully soaked in 8% Na_2_SO_4_ solution with an 18:3 drying–wetting ratio possessed lower compressive strength (Exposure 2, 61.3 MPa) compared to that with a drying–wetting ratio of 9:12 (Exposure 3, 66.3 MPa) and 3:18 (Exposure 1, 77.3 MPa). Exposure 1, which had the shorter drying time, showed the greatest strength of the concrete, which even increased with the number of corroding days. During the drying–wetting cycles, the drying time played an important role in the sulfate deterioration of the SCRPC. Lower saturation during the drying period results in higher capillary suction during the wetting process, which transfers more erosion solution into the matrix [49]. Therefore, the SCRPC soaked in the sulfate solution with a larger drying–wetting ratio was more extensively damage. In addition, no significant compressive strength change could be observed in the SCRPC specimens with semi-soaking and full soaking, which was similar to the mass loss results.

### 3.5. Effective Porosity

**Figure 12:**
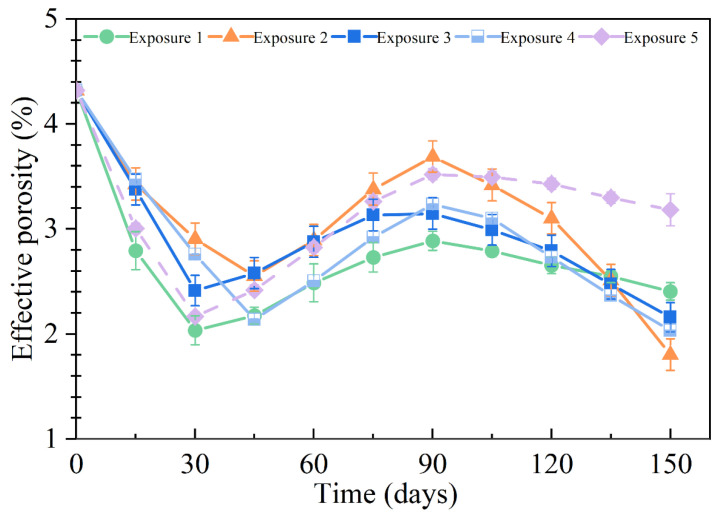
Effective porosity of the SCRPC specimens under different sulfate drying–wetting exposure regimes.

**Figure 13:**
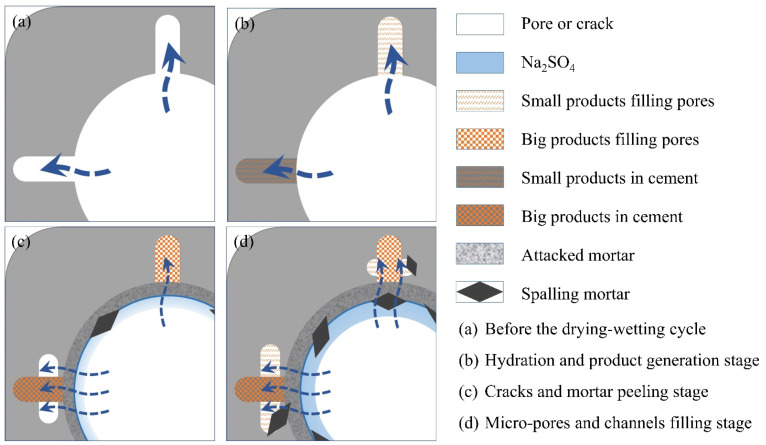
The mechanism of the artificial channel of SCRPC under a Na_2_SO_4_ drying–wetting cycle. (**a**) Before the drying-wetting cycle; (**b**) Hydration and product generation stage; (**c**) Cracks and mortar peeling stage; (**d**) Micro-pores and channels filling stage.

Figure 12 shows the effective porosity of the SCRPC specimens under different sulfate drying–wetting exposure regimes. The effective porosity variation of SCRPC under drying–wetting cycles consists of three stages: an initial rapid decline stage, then a slight rising stage, and finally a slow decline stage. This behavior differs from traditional concrete, which typically exhibits two stages [50]. The hydration of the unhydrated cementitious material and the filling of micropores by expansion products reduced the effective porosity of the SCRPC in the early stages of the sulfate drying–wetting cycles. The accumulation of aggressive products caused additional microcracks as the sulfate drying–wetting cycles progressed, and the surface mortar was attacked to peel off, increasing the effective porosity. The third stage of the drying–wetting cycle in SCRPC was different from traditional concrete [50]. The physical crystallization of Na_2_SO_4_ accumulated in the artificial channel of the SCRPC, decreasing the effective porosity. Furthermore, the peeling mortar around the artificial pore could block the channel [51]. The mechanism diagram of the artificial channel of the SCRPC under a Na_2_SO_4_ drying–wetting cycle is shown in Figure 13.

In the case of the specimens with varying drying–wetting ratios, the specimen under Exposure 1 (drying–wetting ratio of 3:18) showed the greatest decrease in effective porosity at the start of the drying–wetting cycles, followed by Exposure 3 (drying–wetting ratio of 9:12) and Exposure 2 (drying–wetting ratio of 18:3). Longer soaking times facilitated the hydration process of unhydrated cement particles and the initial decrease in effective porosity. After 150 drying–wetting cycles, the effective porosity values of the SCRPC were 2.8%, 1.8%, and 1.7%, respectively, under Exposure 1, Exposure 2, and Exposure 3. A more considerable effective porosity decreasing stage and blocking effects were noted in the SCRPC with a higher sulfate drying–wetting ratio. In addition, the effective porosity of SCRPC with semi-soaking (Exposure 4) was slightly lower than that with full soaking (Exposure 3). In the case of MgSO_4_ solution, the third stage of declining effective porosity was not obvious, which could be due to the inconspicuous crystallization phenomenon of MgSO_4_ during drying–wetting cycles [31].

### 3.6. Permeability Coefficient

**Figure 14:**
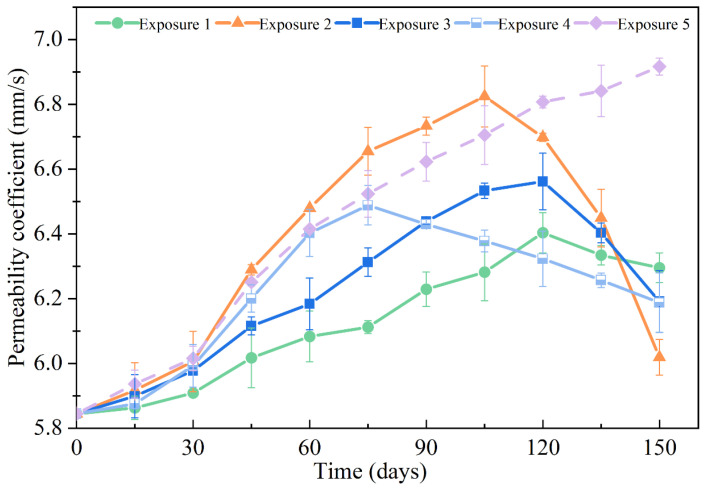
Permeability coefficient of SCRPC specimens under different sulfate drying–wetting exposure regimes.

Figure 14 shows the permeability coefficient of the SCRPC specimens under different sulfate drying–wetting exposure regimes. The permeability coefficient variation of SCRPC can be regarded as having two stages: an initial increasing stage and a subsequent decreasing stage. Generally, the variation in the permeability coefficient is opposite to that of porosity for traditional pervious concrete [52]. However, for the designed SCRPC, the permeability coefficient was mainly determined by the artificial channel. The early expansion of the recycled self-compacting concrete matrix caused by sulfate had little effect on the permeability of the SCRPC. As the sulfate drying–wetting cycles progressed, the expansive deterioration products led to microcracks and increased the permeability coefficient of the SCRPC. At the later stage of the sulfate drying–wetting cycles, the peeling mortar and the accumulating crystals resulted in the blockage of artificial channels, which slightly decreased the permeability coefficient of the SCRPC.

Figure 14 shows that under Exposure 1 (drying–wetting ratio of 3:18), Exposure 2 (drying–wetting ratio of 18:3), and Exposure 3 (drying–wetting ratio of 9:12), the peak permeability coefficients of the SCRPC were 6.40 mm/s, 6.82 mm/s, and 6.56 mm/s, respectively. With the increase in drying–wetting ratio, the peak value of the SCRPC permeability coefficient increased, indicating more microcracks during the sulfate attack. After 150 drying–wetting cycles, the permeability coefficients were 6.27 mm/s, 6.00 mm/s, and 6.17 mm/s, respectively, under Exposure 1, Exposure 2, and Exposure 3. This phenomenon is the opposite to that of the peak permeability coefficient of SCRPC, which could be due to the more obvious blockage caused by mortar peeling.

In addition, it was found that the permeability coefficient of the SCRPC with semi-soaking (Exposure 4) after 150 drying–wetting cycles was nearly the same as that of the SCRPC with full soaking (Exposure 3). The exposure regimes (semi-soaking or full soaking) did not impact the permeability coefficient of the SCRPC during sulfate drying–wetting exposure. The permeability coefficient of the SCRPC under Exposure 5 (MgSO_4_ solution) increased with the number of drying–wetting cycles. As the sulfate drying–wetting cycles progressed, the crystallization pressure of MgSO_4_ was significantly lower than that of Na_2_SO_4_ [53]. Thus, the artificial channel blockage due to crystalline precipitation and the decrease in the permeability coefficient disappeared. Moreover, the permeability coefficients of all the SCRPC specimens exceeded 3.5 mm/s, which meets the requirement of CJJ/T 135-2009 [38]. The designed SCRPC had an excellent water permeability performance after 150 sulfate drying–wetting cycles.

### 3.7. XRD Analysis

**Figure 15:**
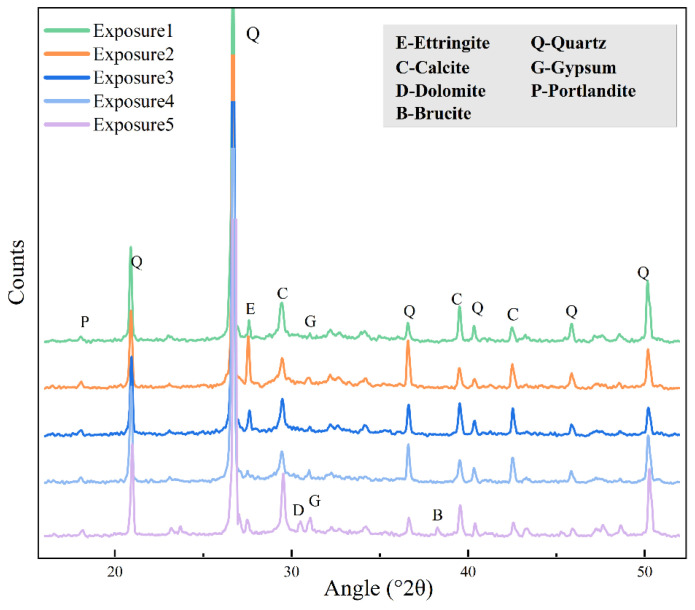
XRD diffractograms of SCRPC specimens under different sulfate drying–wetting exposure regimes.

Figure 15 shows the XRD diffractograms of the SCRPC specimens under different exposure regimes with sulfate drying–wetting cycles. In Figure 15, the maximum diffraction peak of ettringite at 27.56° 2θ is noted in the specimen under Exposure 2 (drying–wetting ratio of 18:3), while the ettringite diffraction peak is relatively lower for the specimen under Exposure 1 (drying–wetting ratio of 3:18). For a longer soaking time in the drying–wetting cycles, the diffusion of sulfate ions was slower, which produced less ettringite. Increasing the drying time could produce more ettringite, which generated more sulfate crystallization pressure and accelerated the diffusion of sulfate ions.

In the case of the semi-soaking under Exposure 4, the ettringite diffraction peak was relatively small. However, the gypsum diffraction peak could be observed clearly at 31.00° 2θ. Although the sulfate resistance of the SCRPC under semi-soaking and full soaking was similar, the main sulfate deterioration products were different. Ettringite was the main sulfate deterioration product of the SCRPC under full soaking, while gypsum was the main sulfate deterioration product of SCRPC under semi-soaking.

In the case of MgSO_4_ solution soaking (Exposure 5), the diffraction peaks of dolomite (CaMg(CO_3_)_2_) and brucite (Mg(OH)_2_) were observed at 30.50° 2θ and 38.26° 2θ, respectively [53]. The gypsum and ettringite diffraction peaks were also observed, which are also important deterioration products of MgSO_4_ attack.

### 3.8. Micromorphology from SEM

**Figure 16:**
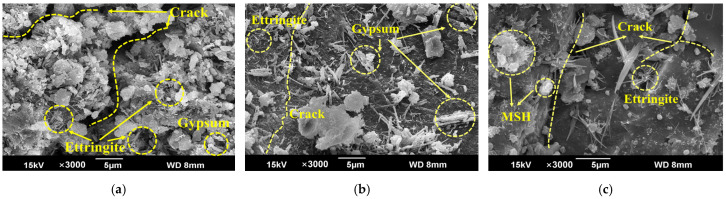
SEM images of SCRPC specimens under different sulfate drying–wetting exposure regimes: (**a**) Exposure 3; (**b**) Exposure 4; (**c**) Exposure 5.

Figure 16 shows the SEM images of the SCRPC specimens under different sulfate drying–wetting exposure regimes. It can be observed that the crack in the SCRPC under Exposure 3 was wide and deep (Figure 16a). Acicular ettringite and short column gypsum products existed at the edge and inside of the crack. Figure 16b shows the SEM images of the upper half of the specimen treated with semi-soaking (Exposure 4). Compared with fully soaked specimens, the products of the semi-soaked specimen were stacked on the surface. Figure 16c shows the unique deterioration product M-S-H of the SCRPC specimen exposed to MgSO_4_. Under Exposure 5, the form of ettringite was filamentous rather than acicular, owing to Mg^2+^ making the ettringite crystal appear fine and small. Compared with Exposure 3, the ettringite accumulated on the M-S-H and bulge parts.

## 4. Conclusions

This study innovatively proposed a self-compacting recycled pervious concrete (using 100% recycled coarse aggregate) with artificial preparation of straight holes. This concrete has excellent sulfate resistance and water permeability properties under different drying–wetting exposures. The main conclusions of this research can be summarized as follows.

1. The maximum mass loss and compressive strength loss of the SCRPC after 150 drying–wetting cycles were found to be only 1.82% and 13.4%, respectively, which occurred under Exposure 5 (MgSO_4_ solution).
2. As the drying–wetting ratio increased, the sulfate degradation of the SCRPC increased. The SCRPC soaked in Na_2_SO_4_ solution with a larger drying–wetting ratio of 18:3 showed a lower RDME (90.6%). During the drying stage, the pressure resulting from supersaturated sulfate crystallization was more deleterious than the expansive products generated during the wetting stage.
3. Although the RDME values of the SCRPC after full soaking (95.54%) and semi-soaking (92.89%) were comparable, the predominant sulfate deterioration products differed. Ettringite and gypsum were the main deterioration products under full soaking and semi-soaking, respectively.
4. The variation in the effective porosity of SCRPC under sulfate drying–wetting cycles can be divided into three stages, different from traditional concrete which typically exhibits two stages. The hydration of unhydrated cementitious materials and the creation of expansive products caused the first rapid decline stage. Microcracks caused the subsequent increasing stage, and the filling of salt crystals and exfoliated mortar to the channels caused the final slow decline stage.
5. The permeability coefficient of the SCRPC varied from 6.00 to 6.82 mm/s, greater than the requirement (3.5 mm/s) for pervious concrete, indicating excellent permeability.

The primary limitation of the current study is the use of only one proportion of concrete. It remains uncertain whether the outstanding sulfate resistance and permeability of the concrete can be sustained through alterations in the mix design or a reduction in strength. Additionally, when pervious concrete is applied in road projects, it is frequently exposed to the combined influences of loading, erosion, and heavy rainfall. Therefore, further research will be conducted on the RSPRC under complex environmental conditions at a later stage.

## Figures and Tables

**Table 1:** Chemical compositions of the used cementitious materials (%).

	CaO	SiO_2_	Al_2_O_3_	Fe_2_O_3_	MgO	MnO	K_2_O	TiO_2_	SO_3_	LOI
Cement	67.48	23.49	2.06	2.97	0.50	0.09	0.89	0.17	1.65	1.16
FA	3.8	52.5	28.3	3.67	1.12	0.20	1.7	1.0	1.8	5.95
SF	0.23	86.18	1.07	0.93	0.78	0.13	-	-	0.85	2.62

**Table 2:** Mix proportion of SCRPC (kg/m^3^).

Cement	Sand	RCA	FA	SF	SP	Water
397	784	840	153	61	4.88	205

**Table 3:** The specified details of sulfate drying–wetting exposure.

No.	Drying–Wetting Ratio	Soaking Method	Solution	Total Cycle
Exposure 1	3:18	Full soaking	8% Na_2_SO_4_	Total 150 cycles (150 days), with one cycle lasting 24 h.
Exposure 2	18:3	Full soaking	8% Na_2_SO_4_	
Exposure 3	9:12	Full soaking	8% Na_2_SO_4_	
Exposure 4	9:12	Semi-soaking	8% Na_2_SO_4_	
Exposure 5	9:12	Full soaking	8% MgSO_4_	

## Data Availability

The original contributions presented in this study are included in the article. Further inquiries can be directed to the corresponding author.
